# The impact of intention to adopt generative AI for exercise information on exercise adherence among Chinese college students: the mediating role of autonomous motivation and network analysis

**DOI:** 10.3389/fpubh.2026.1754288

**Published:** 2026-02-13

**Authors:** Hao Gou, Qunqun Sun, Luyao Xiang, Chang Hu, Yuan Fang, Faxiang Fan

**Affiliations:** 1 College of Physical Education, Qiannan Normal University for Nationalities, Duyun, China; 2 Zhuhai Campus, Zunyi Medical University, Zhuhai, China; 3 College of Physical Education, Jiangxi Normal University, Nanchang, China

**Keywords:** autonomous motivation, exercise adherence, generative artificial intelligence, intention to adopt exercise information, mediation effect, network analysis

## Abstract

**Background:**

Insufficient exercise adherence among college students is a common health issue, while generative artificial intelligence (AI) provides a new approach for personalized exercise guidance. However, the extent to which the intention to adopt generative AI for exercise information affects exercise adherence, and the role of autonomous motivation in this process, remains underexplored in empirical research.

**Methods:**

This study employed a cross-sectional survey design and administered a questionnaire to 1,878 Chinese undergraduates *via* stratified random cluster sampling. The core variables were measured using the self-developed “Intention to Adopt Generative AI for Exercise Information Scale,” the Chinese version of the “Autonomous Motivation Scale,” and the “Exercise Adherence Scale.” Mediation effects were tested using the PROCESS macro, and network analysis was performed in R to examine the complex interactions among variables.

**Results:**

The intention to adopt generative AI for exercise information was significantly positively correlated with exercise adherence among college students (*β* = 0.299, *p* < 0.001), and it produced a significant indirect effect by enhancing autonomous motivation (effect size = 0.116, 95% CI [0.094, 0.139]), with the mediation effect accounting for 27.78% of the total effect. Network analysis further identified “intrinsic motivation” and “behavioral habits” as the core nodes with the most significant influence on the overall psychological-behavioral network.

**Conclusion:**

This study explores the associations and potential mechanisms by which generative AI may relate to exercise adherence via autonomous motivation, supporting a theoretical pathway of “technology adoption—motivation internalization—behavior persistence.” The findings offer a novel perspective on the theoretical associations underlying AI-enabled health behaviors and provide preliminary correlational evidence to inform the future design of generative AI applications for health interventions.

## 1 Introduction

In recent years, artificial intelligence (AI) technology, particularly generative AI, has been rapidly permeating and reshaping various aspects of social life (1). Compared with traditional information retrieval and analysis tools, generative AI, with its robust natural language understanding and content generation capabilities, can dynamically and creatively deliver highly customized information and solutions tailored to users’ personalized needs (2, 3). This interactive and intelligent feature has shown enormous potential in areas such as knowledge Q&A, content creation, and learning assistance, quickly becoming an essential tool for the younger generation, especially college students, to explore the world and acquire new knowledge (4–6). Surveys indicate that college students are among the most active groups in terms of exposure to and use of generative AI, with its use remaining high (7, 8). They primarily use this technology to support academic research, spark creative inspiration, optimize learning processes, and access daily information (9).

Recent studies have highlighted the growing importance of AI in health promotion, particularly in digital health interventions. AI-driven tools are increasingly being employed to support personalized health behavior change, such as encouraging physical activity and providing tailored health advice. These interventions not only enhance engagement but also empower individuals to take control of their health, fostering long-term behavioral changes (10). In the realm of exercise, AI has shown significant promise, particularly by providing contextually relevant, real-time guidance that adapts to users’ needs (11). Such interventions, driven by AI, align well with the need for personalized exercise programs, especially in populations like college students who struggle with exercise adherence due to factors such as time constraints and motivation. This shift toward AI-enabled health promotion has the potential to transform the landscape of exercise behavior, offering scalable, accessible, and personalized solutions. Despite the exciting possibilities, empirical research exploring the specific impact of generative AI on exercise adherence remains limited (12). While the role of AI in broader healthcare contexts is well established, its application in promoting sustained exercise habits, especially among university students, remains underexplored (13). College students are at a critical stage in forming lifestyle habits while facing academic pressure and fragmented time (14).

Effectively maintaining regular exercise has become a common challenge (15). If generative AI can provide scientifically accurate, contextually relevant exercise guidance, it could become an effective tool to help college students engage in scientific exercise and enhance their exercise experience (16). However, there is currently a lack of systematic empirical research on whether college students are willing to adopt generative AI for exercise information, how they adopt it, and how this adoption intention influences their actual exercise adherence (17, 18). Therefore, this study focuses on the core concept of Intention to Adopt Generative AI for Exercise Information (IAGEI) and aims to explore its impact mechanism on Exercise Adherence (EA) among college students. Guided primarily by self-determination theory, which posits that sustained behavior change is facilitated by autonomous motivation arising from supports for basic psychological needs, we investigate whether IAGEI is associated with EA via the mediating role of Autonomous Motivation. Complementary perspectives from the Technology Acceptance Model and the Theory of Planned Behavior inform the conceptualization of IAGEI and its initial link to behavior. We further employ network analysis to explore nuanced interactions among specific dimensions of these constructs, aiming to provide new theoretical perspectives and practical insights into how digital technologies can empower health behavior promotion.

### 1.1 The relationship between IAGEI and EA

The IAGEI broadly refers to an individual’s psychological tendency and behavioral readiness to use generative AI tools (such as large language models) to search for, consult, and accept exercise-related guidance, plans, or suggestions generated by the AI (19). In this study, this concept is operationalized as a multidimensional construct. It not only refers to the frequency at which college students use such technologies but also, more importantly, encompasses the breadth of the types of exercise information they seek (e.g., personalized plans, movement correction, nutritional advice, etc.), as well as their perceptions of and trust in the quality of the information they receive, ultimately reflecting their intrinsic willingness to continue using and recommend the tool to others (20, 21).

Critically, IAGEI pertains specifically to engagement with generative AI, which represents a qualitative shift from traditional digital exercise platforms (22). Unlike static information repositories, rule-based fitness applications, or algorithmic recommenders operating within fixed parameters, generative AI introduces distinct intervention mechanisms (23). Its core affordances include open-ended dialogic interaction, allowing for multi-turn conversations that understand nuanced user constraints; adaptive reasoning and content generation, synthesizing contextualized plans rather than retrieving static information; and the capacity to simulate key coaching functions such as empathetic encouragement and collaborative goal-setting (24). Therefore, IAGEI captures the intention to engage with a tool that functions as an interactive, reasoning partner, making it a theoretically distinct precursor to behavior change compared to intentions toward more passive or rigid digital tools (25).

According to social cognitive theory, a positive intention to adopt information often indicates a deeper process of interaction and integration with the information (26), whereas, according to the theory of planned behavior, a firm behavioral intention is a key antecedent of actual behavior (27). Existing studies have shown that convenient, accurate, and high-quality exercise information support can enhance an individual’s exercise self-efficacy, reduce cognitive and behavioral barriers to starting and maintaining exercise, and thus positively impact sustained regular exercise (28–30). Compared with traditional digital exercise information sources such as fixed-function fitness apps or pre-recorded online tutorials, generative AI can engage in open-ended natural language interaction, flexibly adjusting the content, difficulty, and framing of its suggestions in response to users’ continuous feedback in real time (31). This dialogic, co-constructed form of guidance allows users to negotiate exercise goals, constraints, and preferences with the system, which may strengthen their perceived behavioral control and intention in line with the theory of planned behavior, beyond what is typically achieved by one-way information delivery channels (32). At the same time, the capacity of generative AI to simulate the functions of a virtual coach—for example by offering encouragement, reframing setbacks, and iteratively refining exercise plans—may create a qualitatively different motivational climate from that of static information platforms, thereby making it a potentially novel type of technology-enabled intervention rather than merely a new delivery medium for existing content (33). Based on this, this study hypothesizes that IAGEI will positively predict EA.

### 1.2 The relationship between AM, IAGEI, and EA

This study is principally grounded in self-determination theory (34), which provides the core mechanism—autonomous motivation—for understanding sustained behavioral engagement. AM, derived from self-determination theory (35), refers to the psychological quality of engaging in a behavior for intrinsic reasons, such as interest, enjoyment, or value alignment, rather than external pressure or rewards. It is a key predictor of behavioral persistence (36). On one hand, according to the “basic psychological needs” sub-theory within self-determination theory, individuals have fundamental psychological needs for autonomy, competence, and relatedness (34). Generative AI, by providing highly personalized and interactive exercise information, can directly support users’ autonomy feeling that the behavior is self-chosen and competence feeling capable of effectively executing plans (34, 37). The satisfaction of these needs is a crucial situational factor that promotes the internalization of external motivation and enhances levels of AM (38). Compared with conventional digital tools that provide pre-set exercise plans, generative AI can engage in iterative question–answer cycles in which students explain their situational constraints, receive tailored suggestions, and immediately ask follow-up questions, thereby allowing them to experience a stronger sense of authorship over the final plan (39). From a self-determination theory perspective, this co-creation process may be especially conducive to autonomy-supportive communication and to the internalization of exercise goals, which helps explain why the present model emphasizes intention to adopt generative AI, rather than generic exposure to digital exercise content, as the starting point (40). On the other hand, both self-determination theory itself and a wealth of empirical research have clearly pointed out that compared to controlled motivation (35), AM can significantly predict the long-term adherence to various health behaviors, as it is closely linked to higher enjoyment of behavior, stronger psychological vitality, and greater perseverance in the face of setbacks (41). IAGEI may create an information environment that supports basic psychological needs, which in turn nourishes individuals’ AM, and this high-quality motivation drives sustained exercise behavior (42). Therefore, this study hypothesizes that AM plays a significant mediating role in the relationship between IAGEI and EA.

### 1.3 Introducing network analysis: unveiling the deep interactions among variables

To complement conventional variable-centered analyses and uncover the complex system of interdependencies among specific psychological and behavioral dimensions, this study employs network analysis (43, 44). This method moves beyond examining relationships between latent constructs to model the web of pairwise associations among observable dimensions (45, 46). Its value lies in its capacity to reveal which specific facets of constructs (e.g., which dimension of motivation or adherence) are most centrally connected within the observed system, offering a nuanced map of the psychological-behavioral architecture (47). Building on this, we construct a network model among the dimensions of IAGEI, AM, and EA. The goal is not to infer causality but to achieve a more granular understanding: First, to reveal which specific IAGEI characteristics show the strongest conditional associations with which types of AM and EA dimensions (48); Second, to exploratorily identify nodes (dimensions) with high connective influence, which may represent promising focal points within the associative network for future hypothesis-driven research (49). Thus, the network analysis serves as a valuable exploratory supplement (50), providing a detailed visualization of the systemic interrelations that can inform future theoretical refinement and intervention design (51).

**Figure 1:**
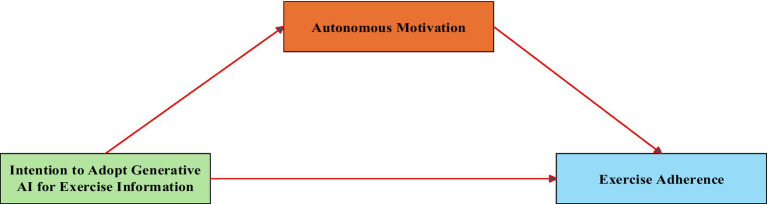
Hypothetical model.

Figure 1 illustrates the conceptual model depicting the hypothesized relationships among these variables.

## 2 Methods

### 2.1 Participants and procedures

From April to June 2025, an online survey was conducted using stratified random cluster sampling. One undergraduate university was randomly selected from each of four provinces: Guizhou, Jiangxi, Guangdong, and Henan. In each university, four classes were randomly selected at each grade level, totaling 48 classes and 48 classes of students as survey participants. All students in the selected classes were invited to participate in the online questionnaire survey. All participants provided informed consent and voluntarily agreed to participate. The inclusion criteria for participants were: (1) ability to understand and cooperate with the survey; (2) voluntary participation in the study. Exclusion criteria included: (1) incomplete questionnaire responses (less than 80% completion); (2) refusal to participate in the survey. A total of 1,968 questionnaires were collected. After removing invalid questionnaires due to patterned responses and failure to pass lie-detection questions, the missing values in the remaining data were handled using multiple imputation via chained equations (MICE) (52). The imputation was conducted using the mice package in R. Ultimately, 1,878 valid questionnaires were obtained, yielding an effective response rate of 95.4%.

Among the 1,878 college students included in the analysis (as shown in Table 1), the distribution of their demographic characteristics is as follows: 752 males (40.0%) and 1,126 females (60.0%); grade distribution included 537 first-year students (28.6%), 576 s-year students (30.7%), 414 third-year students (22.0%), and 351 fourth-year students (18.7%); regarding their residence, 795 students (42.3%) lived in rural areas, while 1,083 students (57.7%) lived in urban areas.

**Table 1:** Basic characteristics of the sample.

Variable	Sort	Frequency	Scale
Sex	Male	752	40.00%
	Female	1,126	60.00%
Grade	First year	537	28.60%
	Second year	576	30.70%
	Third year	414	22.00%
	Fourth year	351	18.70%
Residence	Rural	795	42.30%
	Urban	1,083	57.70%

### 2.2 Measuring tools

#### 2.2.1 Exercise adherence scale

In this study, the Exercise Adherence Scale developed by Wang Shen’s team was used to assess participants’ exercise adherence (53). The scale consists of 14 items, covering three dimensions: behavioral habits, effort investment, and emotional experience. It uses a 5-point Likert scale (1 = Strongly disagree, 5 = Strongly agree), with higher total scores indicating stronger exercise adherence. The empirical data showed that the Cronbach’s *α* coefficient for the overall scale was 0.898. Confirmatory factor analysis revealed a good model fit: χ^2^/df = 1.818, RMSEA = 0.021, SRMR = 0.013, CFI = 0.996, TLI = 0.995. The Cronbach’s α for the total scale was 0.887, and the α coefficients for each dimension ranged from 0.870 to 0.901.

#### 2.2.2 Autonomous motivation scale

In this study, the Behavioral Regulation Scale was developed by Liu et al. (54) was used, and it was culturally adapted to Chinese through a standard procedure: first, two researchers independently completed a forward translation and synthesized the initial version; then, a translator who had not seen the original scale performed a back-translation; finally, the research team and experts compared the back-translated version with the original to conduct cultural adjustments, resulting in the final Chinese version. The scale consists of 14 items, divided into four dimensions: external regulation (4 items), introjected regulation (3 items), identified regulation (3 items), and intrinsic motivation (4 items). A 7-point Likert scale was used (1 = “Strongly disagree,” 7 = “Strongly agree”). Confirmatory factor analysis showed a good model fit: *χ^2^/df* = 1.587, *RMSEA* = 0.018, *SRMR* = 0.011, *CFI* = 0.997, *TLI* = 0.997. The *α* coefficients for each dimension ranged from 0.864 to 0.899.

#### 2.2.3 Self-developed intention to adopt generative AI for exercise information scale

At the initial stage of scale development, this study integrated the Unified Theory of Acceptance and Use of Technology (UTAUT) (55), Perceived Fit Theory (56), and the classic Attitude-Behavior Theory (57). It is closely aligned with the strategic deployments under “empowering national fitness through digital technologies” in the “Healthy China 2030 Plan” and the “14th Five-Year Plan for Sports Development.” The study initially established four core dimensions of generative AI exercise information adoption behavior: perceived usefulness, perceived fit, attitude toward use, and adoption intention. Specifically, “perceived usefulness” refers to the college students’ subjective evaluation of whether the exercise information provided by generative AI can effectively help them improve their exercise outcomes. “Perceived fit” emphasizes the degree to which students believe generative AI can provide highly personalized exercise plans based on their individual physical condition, goals, equipment, and time constraints. “Attitude toward use” reflects the positive or negative emotional tendency that college students have toward the behavior of using generative AI to obtain exercise information. “Adoption intention” directly measures students’ willingness to use the technology in the future and to recommend it to others.

To construct an indicator pool that closely aligns with users’ real experiences, this study employed grounded theory to conduct semi-structured, in-depth interviews with 30 college students who regularly exercise and had previously used generative AI. The interview topics covered specific scenarios in which generative AI was used to address exercise and health issues, the evaluation of the logic and depth of AI-generated content, strategies for optimizing instructions across multiple Q&A sessions, evaluation of information credibility, and concerns about privacy and security, among others.

Through three levels of analysis—open coding, axial coding, and selective coding of the interview texts—we extracted four core categories (first-level dimensions), eight main categories (second-level indicators), and 16 concepts (third-level concepts). Based on this, we preliminarily developed a theory-driven item pool. Specific examples are as follows: For the “perceived usefulness” dimension, we identified “expected effectiveness” and “efficiency improvement” as the second-level indicators. From interview statements (e.g., “I asked AI how to train chest muscles effectively, and it gave me a complete plan, explaining the principles. This was much more useful than searching scattered information online”), we abstracted concepts such as “goal achievement expectations” and “perceived value of information.” Based on these, we drafted items PU1 and PU3. For the “perceived fit” dimension, we identified “personalized plans” and “dynamic adjustability” as the second-level indicators. From interview statements (e.g., “I told AI I only had dumbbells, and it generated a dumbbell-only plan. I said the last plan was too tiring, and it immediately adjusted the intensity for me”), we abstracted concepts such as “precise plan generation” and “feedback optimization ability,” and based on these, we drafted items PF1 and PF3. For the “attitude toward use” dimension, we identified “emotional preference” and “cognitive evaluation” as the second-level indicators. From interview statements (e.g., “Chatting with AI to set up a plan is very fun, like having a personal trainer to help me think. I really like this approach”), we abstracted concepts such as “interaction enjoyment” and “method wisdom,” and based on these, we drafted items ATU2 and ATU1. For the “adoption intention” dimension, we identified “willingness to continue using” and “tendency to share and recommend” as the second-level indicators. From interview statements (e.g., “I’ve gotten used to asking AI before starting new training. I will definitely continue using it in the future and have recommended it to my workout buddy”), we abstracted concepts such as “behavioral dependence” and “active promotion,” and based on these, we drafted items ADI1 and ADI3.

Building on this, the study organized two rounds of Delphi expert consultations, inviting 15 experts from sports science, artificial intelligence, health communication, psychology, and measurement. Based on the criteria of an importance rating mean > 3.50, a coefficient of variation < 0.25, and a response rate> 20%, the initial item pool was iteratively revised and refined. The experts strongly affirmed the dimensional structure and content validity of the items and provided valuable feedback on the precision and accessibility of specific item formulations. For example, the phrase “The plan provided by AI is superior” was revised to “The plan provided by AI is scientific and effective.” Ultimately, we finalized an initial scale consisting of 4 first-level dimensions and 12 specific items.

Through convenience sampling, a pretest was conducted with 418 college students, and valid questionnaires were collected. The following steps were then taken to conduct rigorous item analysis for reliability and validity testing, with detailed tables provided in the appendix:

*Item analysis*: The critical ratio method and item-total correlation method were used. The results showed that the essential values for all items were greater than 0.3, and the correlation coefficients between each item and the total score were all above 0.4, indicating that all items had good discrimination and were retained.

*Reliability analysis*: Internal consistency of the initial scale was tested. The Cronbach’s *α* coefficient for the overall scale was 0.886, and the α coefficients for each dimension were also above 0.80, indicating excellent reliability.

*Exploratory factor analysis (EFA)*: Principal component analysis with varimax rotation was employed. The sample appropriateness test yielded a KMO value of 0.837, and Bartlett’s test of sphericity was significant (*p* < 0.001), indicating that the data were suitable for factor analysis. The analysis extracted four factors with eigenvalues greater than 1, and the cumulative variance explained reached 86.51%. All items had factor loadings greater than 0.4 on their respective factors, and no significant cross-loadings were observed, confirming that the factor structure fully aligned with the theoretical framework.

*Confirmatory factor analysis (CFA)*: A four-factor model was tested in AMOS. The goodness-of-fit indices were as follows: *χ^2^/df* = 1.276, *RMSEA* = 0.026, *SRMR* = 0.036, *CFI* = 0.997, *TLI* = 0.995. All indices met the criteria for a good model fit, and the standardized factor loadings for all items were above 0.60, demonstrating excellent structural validity.

In the subsequent formal survey, the scale’s Cronbach’s *α* coefficient was 0.880, with the α coefficients for each dimension ranging from 0.856 to 0.877. The CFA again confirmed good model fit (*χ^2^/df* = 1.505, *RMSEA* = 0.019, *SRMR* = 0.016, *CFI* = 0.994, *TLI* = 0.993), indicating that the IAGEI Scale has stable reliability and validity and is suitable for formal research.

### 2.3 Data analysis

SPSS 27.0 was used to conduct item discrimination tests, item-total correlation analysis, and exploratory factor analysis on the 418 pre-survey samples. Subsequently, AMOS 26.0 was used to perform a confirmatory factor analysis to assess the structural validity and reliability of the IAGEI scale. In the formal survey, 1,878 valid questionnaires were analyzed using SPSS 27.0 with the PROCESS macro (model 4) for mediation analysis, with gender, age, grade, and residence as covariates. The network analysis was performed in R 4.3.2, using a mixed graphical model for estimation and applying the EBICglasso algorithm for regularization to streamline the network structure. The hyperparameter *γ* for the extended Bayesian information criterion (EBIC) was set to 0.5. For node centrality evaluation, the expected influence was chosen as the measure of node impact, as it shows good stability across mixed-polarity networks. Finally, the Bootstrap sampling method was used for 1,000 iterations to calculate 95% confidence intervals for edge weights to assess estimation accuracy and test the stability of centrality indicators. The acceptable stability coefficient (CS) was set to CS > 0.25, with CS > 0.5 indicating good stability.

## 3 Results

### 3.1 Common variance test

This study used Harman’s single-factor test to analyze common method bias. The results showed that 11 factors had eigenvalues greater than 1, with the first factor accounting for 21.24% of the total variance. Since this value is below the 40% threshold, it indicates that there is no significant standard method bias in the data of this study.

### 3.2 Correlation analysis

A correlation analysis was conducted on the means of IAGEI, EA, and AM. The results showed that IAGEI, EA, and AM were positively correlated with each other (as shown in Table 2).

**Table 2:** Means, standard deviations, and correlation coefficients of each variable.

Variables	M ± SD	IAGEI	EA	AM
IAGEI	3.06 ± 0.94	1		
EA	3.07 ± 0.92	0.414***	1	
AM	4.05 ± 1.38	0.362***	0.428***	1

*p < 0.05, **p < 0.01, ***p < 0.001. The same notation applies below.

### 3.3 Mediation effect of AM

Using SPSS 27.0 and PROCESS 4.1 macro (Model 4), IAGEI was set as the independent variable, EA as the dependent variable, and AM as the mediator to test the mediation effect of AM. Gender, age, grade, and residence were included as covariates in the model. The results (Table 3) showed that IAGEI was significantly positively correlated with AM (*β* = 0.362, *p* < 0.001), and AM also significantly predicted EA (*β* = 0.320, *p* < 0.001). After including AM in the model, IAGEI still showed a positive association with EA (*β* = 0.299, *p* < 0.001). The Bootstrap test showed that the indirect effect of AM was 0.116, with a 95% confidence interval that did not include zero, indicating a statistically significant association consistent with mediation. This cross-sectional finding supports the hypothesized theoretical model in which AM plays a mediating role. As shown in Table 4. This effect accounted for 27.78% of the total effect.

**Table 3:** Regression analysis between variables.

Dependent variable	Independent variable	*R*	*R^2^*	*F(df)*	*β*	*t*
AM	IAGEI	0.363	0.132	56.971	0.362	16.791
	Sex				−0.045	−1.009
	Age				−0.014	−0.715
	Grade				0.001	0.092
	Residence				−0.028	−0.641
EA	IAGEI	0.512	0.262	110.533	0.299	14.005
	AM				0.320	15.002
	Sex				0.024	0.598
	Age				−0.010	−0.528
	Grade				−0.003	−0.303
	Residence				−0.054	−1.348

**Table 4:** Mediation effect of AM.

Effect	Effect size	Bootstrap standard error	95% lower	95% upper	Effect size (%)
Total	0.414	0.021	0.373	0.456	
Direct	0.299	0.021	0.257	0.341	72.22%
Indirect	0.116	0.012	0.094	0.139	27.78%

### 3.4 Network analysis

**Figure 2:**
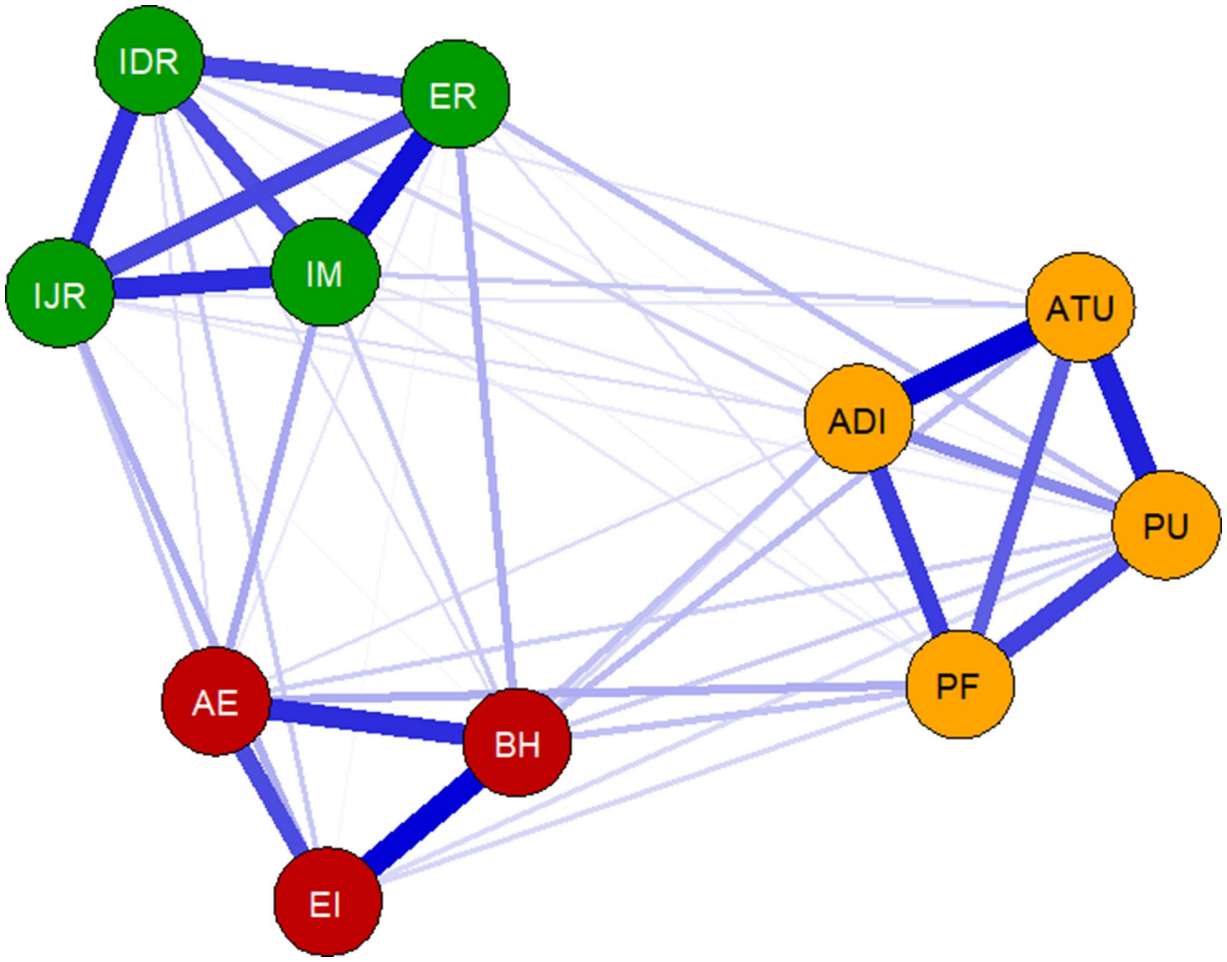
Cross-sectional network structure. Nodes represent variables, and edges represent partial correlations between variables. Green edges indicate positive correlations, and red edges indicate negative correlations. The thickness of the edges is proportional to the strength of the correlation. The variables included in the network are Perceived usefulness (PU), Perceived fit (PF), Attitude toward use (ATU), Adoption intention (ADI), Behavioral habit (BH), Effort investment (EI), Affective experience (AE), External regulation (ER), Introjected regulation (IJR), Identified regulation (IDR), and Intrinsic Motivation (IM).

This study used network analysis to explore the associative structure between the dimensions of IAGEI, AM, and EA. The network estimation results showed that the network contained 54 non-zero edges, with a network density of 0.98, and a non-zero edge proportion of 98.18%, indicating a highly interconnected system among the observed variables. In the network structure (as shown in Figure 2), the strongest connections within dimensions were found in the following relationships: EA’s “Behavioral Habits (BH)” and “Emotional Experience (EI)” were most strongly related (*r* = 0.252), followed by IAGEI’s “Attitude toward Use (ATU)” and “Adoption Intention (ADI)” (*r* = 0.250). In terms of cross-dimensional associations, two key connections are noteworthy: EA’s “Emotional Experience (AE)” and AM’s “Intrinsic Motivation (IM)” showed a significant positive correlation (*r* = 0.085), and IAGEI’s “Perceived Fit (PF)” and EA’s “Emotional Experience (AE)” also showed a close connection (*r* = 0.080). The network analysis results suggest that positive emotional experiences are not only strongly related to intrinsic motivation but are also significantly influenced by the degree of personalized information fit, forming a complex interaction network among the variables. Detailed values of all edges in the cross-sectional network are provided in Supplementary Table S5.

**Figure 3:**
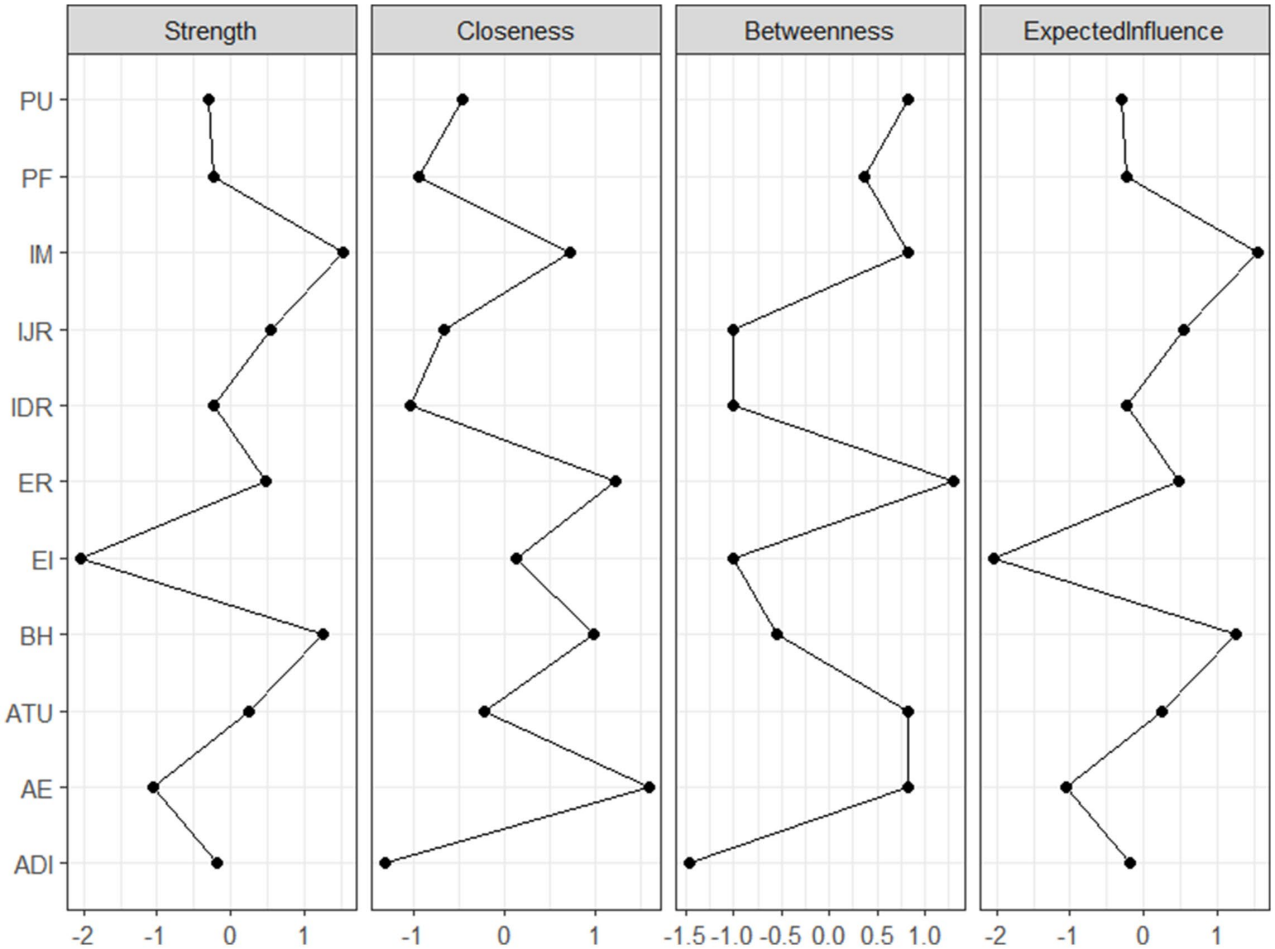
Expected influence (EI) centrality indices of cross-sectional networks.

The centrality analysis results (Figure 3) show that Intrinsic Motivation (IM) under the AM dimension (EI = 1.537) and Behavioral Habit (BH) under the EA dimension (EI = 1.245) are the two nodes with the highest expected influence values. This suggests that motivation driven by intrinsic interest and enjoyment, as well as a regularized exercise behavior pattern, may play the most central driving roles in the entire psychological-behavioral system, having the most substantial overall influence on other variables. Supplementary Table S6 provides detailed centrality indicators for all nodes at two time points.

To assess the reliability of the estimated network, this study used the Bootstrap sampling method (1,000 iterations) to test the stability of the network structure and centrality indicators. The results showed that the stability coefficients (CS-coefficient) for edge weights and shortest path distances were both 0.75, indicating that the estimation of network connectivity strength is very stable. Regarding node centrality indicators, the stability coefficients for expected influence and strength were both 0.361, which are above the acceptable threshold of 0.25, suggesting that the centrality ranking of core nodes is reliable. Overall, the network model in this study demonstrates good stability in estimating edge weights and key influential nodes, supporting subsequent analysis and discussion. The Bootstrap confidence intervals for edge weights and centrality difference test results are provided in the Supplementary materials.

## 4 Discussion

### 4.1 The impact of IAGEI on EA

This study confirmed that IAGEI has a significant positive correlation with EA. This result is consistent with the findings of a cross-sectional study (58). The theory of planned behavior provides a theoretical foundation for understanding this relationship (27), emphasizing that behavioral intention is the most direct predictor of actual behavior (59). Once college students form a stable IAGEI, their psychological readiness to incorporate the technological tool into their exercise planning may transform into sustained information inquiry and plan execution, ultimately enhancing the regularity and persistence of exercise behaviors (60). At the mechanism level, generative AI, by providing highly personalized and instant-feedback exercise plans, effectively addresses the information asymmetry and execution uncertainty that college students face during exercise (61–63). Unlike traditional fixed-mode training plans, AI can dynamically adjust content based on the individual’s real-time status (64). This precise adaptation feature significantly improves the feasibility and acceptance of the exercise plan, further promoting the formation of exercise habits by enhancing self-efficacy (65). Recent research has highlighted the growing importance of AI in promoting physical activity, particularly in improving mental health. For example, a comprehensive review has demonstrated significant evidence supporting the mental health benefits of sustained physical activity, which aligns with our findings on exercise adherence (66). This reinforces the idea that AI-driven interventions could be instrumental in enhancing both physical and mental health by providing personalized, adaptive exercise plans.

Additionally, the increasing role of AI in providing personalized guidance is discussed in recent studies on explainable AI in clinical settings. While these studies primarily focus on clinical decision support, they offer valuable insights into how AI can facilitate user trust and deliver tailored advice (67). These insights are directly applicable to the domain of exercise adherence, where personalized AI-driven solutions are critical for encouraging sustained engagement and adherence to exercise routines. In the context of Chinese higher education, this mechanism is particularly prominent. Faced with intense academic pressure and fragmented time, traditional exercise modes are often challenging to execute effectively (68). Generative AI, with its 24/7 availability and personalized customization, meets the dual needs of flexibility and efficiency among contemporary college students (69). It offers a feasible technological solution for maintaining regular exercise under limited time and space conditions, reflecting the practical value of the Healthy China strategy powered by digital technologies in the campus environment (70–72).

### 4.2 The mediating role of AM between IAGEI and EA

Consistent with our primary theoretical framework (SDT), the cross-sectional mediation analysis indicated a significant associative pattern in which AM links IAGEI and EA. An SDT-informed explanation involves two interrelated phases: motivation formation and behavior maintenance. Generative AI creates an information environment that supports autonomy and competence needs by providing highly personalized exercise plans (73). When students perceive that the AI system can offer “tailored” training recommendations based on their physical fitness level, equipment conditions, and time constraints (74, 75), this precise fit not only enhances the tool’s perceived value but also promotes the internalization of external motivation by respecting individual choice and boosting confidence in execution, thus creating the necessary conditions for the development of AM (76–78).

In the behavior maintenance phase, AM’s promotion of EA reflects the sustained effect of high-quality motivation. Unlike controlled motivation driven by external rewards or punishments, AM, rooted in interest identification and value internalization, can trigger more positive emotional experiences and greater behavioral resilience (79). In the context of Chinese higher education, amid anxiety over credit pressure and social comparisons, exercise driven by AM is more likely to maintain stability because it aligns with students’ intrinsic needs for self-development rather than becoming an additional burden (80, 81). This associative pattern suggests that generative AI may not only be directly linked to exercise behavior by providing information support but, more importantly, fosters high-quality motivation through creating a needs-supportive environment, thus providing intrinsic motivation for behavior persistence (82). This mechanism offers a new perspective for understanding the deeper role of intelligent technologies in promoting health behaviors. Future campus health promotion should focus on cultivating students’ autonomous identification with exercise through technological means, rather than relying solely on external incentives (83, 84).

### 4.3 Network structural features of interactions between variables and intervention implications

The exploratory network analysis provided a detailed map of the associative relationships between the dimensions of IAGEI, AM, and EA. The high connectivity (network density = 0.98) observed in the network model indicates a tightly interrelated system, which may reflect the inherent synergy between these psychological and behavioral constructs in the context of exercise. This systemic perspective complements the variable-centered mediation analysis. The strong internal connections within dimensions reveal the inherent consistency of each psychological construct. The close relationship between EA’s “Behavioral Habit (BH)” and “Affective Experience (AE)” supports the core idea of habit formation theory (85). As exercise behaviors become automated through repeated execution, the conscious effort required diminishes, and the accompanying positive emotional experience further reinforces the behavior’s sustainability (86). This finding is particularly significant among Chinese college students—faced with heavy academic pressures—solidifying exercise into a daily habit that requires no extra willpower and deriving emotional pleasure from it becomes a key factor in maintaining long-term exercise (87, 88). Similarly, the strong connection between “Attitude toward Use (ATU)” and “Adoption Intention (ADI)” in IAGEI supports the basic assumption of the Technology Acceptance Model, where an individual’s positive evaluation of technology directly translates into a willingness to use it (89). Among the cross-dimensional connections, the association between AE and Intrinsic Motivation (IM) enriches self-determination theory by highlighting the interaction between motivation and emotion (35). Positive emotional experiences provide an emotional foundation for cultivating IM by satisfying basic psychological needs, and this mechanism is particularly significant in the context of Chinese culture, which emphasizes the “integration of body and mind” in health concepts (90). At the same time, the connection between Perceived Fit (PF) and AE (*r* = 0.080) suggests that the personalized content provided by generative AI is not only practically valuable but also capable of eliciting positive emotional responses by precisely matching individual needs, offering new evidence for understanding the emotional value of technological fit (91).

The results of the exploratory network centrality analysis show that Intrinsic Motivation (IM) and Behavioral Habit (BH) exhibit the most substantial expected influence (EI values of 1.537 and 1.245, respectively), suggesting they may be influential nodes within the observed associative network. The observed high centrality of IM is consistent with the core idea of self-determination theory (35). As the purest form of AM, IM provides lasting intrinsic motivation for behavior persistence by fulfilling an individual’s needs for autonomy, competence, and relatedness (92). Its position in this cross-sectional network suggests it as a potential candidate for exercise motivation derived from interest and enjoyment, which not only directly influences behavior persistence but also generates ripple effects by activating other psychological processes, such as enhancing emotional experience and promoting cognitive evaluation (93, 94). The high centrality of BH reflects the importance of habit formation theory in EA research. When exercise behavior becomes automated through repeated execution, its demand for cognitive resources decreases, which is particularly valuable in the high cognitive load and fragmented time context of Chinese college students (95). The formation of habits not only directly sustains exercise frequency but also establishes a “behavior-emotion” positive feedback loop through its strong connection to emotional experience, a mechanism particularly prominent in Chinese culture, which emphasizes the integration of knowledge and action (96). In the context of Chinese higher education, the well-known positions of these two core nodes hold significant practical implications. Facing heavy academic pressure and a competitive environment, the primary challenges to college students’ EA have shifted from a lack of knowledge to a lack of motivation and difficulty forming habits (97). IM, by satisfying students’ needs for autonomy and competence, provides intrinsic motivation for EA, while BH helps students overcome willpower depletion through its automation mechanism. Together, they constitute the key psychological resources for addressing the unique challenges faced by Chinese college students.

## 5 Conclusion

Based on cross-sectional associations, this study provides evidence that IAGEI is positively associated with EA, both directly and indirectly through AM, lending support to a theoretical pathway of “technology adoption—motivation internalization—behavior persistence.” Exploratory network analysis further identified Intrinsic Motivation (IM) and Behavioral Habit (BH) as central nodes in the observed associative network, hypothesizing them as potential priority targets for future intervention strategies. These findings not only provide a theoretical explanation of how intelligent technologies empower health behaviors but also offer empirical evidence for implementing precision, motivation-oriented exercise-promotion practices based on generative AI in Chinese higher education contexts.

## 6 Contributions

### 6.1 Theoretical significance

This study makes threefold contributions at the theoretical level: First, it successfully integrates the fields of technology acceptance and health behavior by constructing and validating a theoretical model in which IAGEI serves as the starting point, and AM mediates the process, ultimately influencing EA. This model provides a novel and comprehensive theoretical framework for understanding how digital technologies empower health behavior promotion (98). Second, the study introduces network analysis, an advanced method that goes beyond the traditional variable-centered paradigm. It visually reveals the central roles of Intrinsic Motivation (IM) and Behavioral Habit (BH) within the psychological-behavioral system, confirming the core propositions of self-determination theory and habit formation theory and deepening our understanding of the complex interactions among variables from a system perspective (35). Lastly, this study elevates the role of generative AI from an “information tool” to a “motivation catalyst,” uncovering the deep mechanism through which it cultivates AM by fulfilling basic psychological needs (99). This significantly enriches and expands the application of self-determination theory in the context of intelligent human-machine interactions.

### 6.2 Practical significance

At the practical level, the findings of this study provide a clear roadmap for using artificial intelligence to promote physical exercise among college students in the context of Chinese higher education. The study suggests that educators and health promoters should not merely view generative AI as a “broadcast station” for delivering training plans, but should leverage its personalized and interactive features to transform it into a “motivational partner” that supports students’ autonomous decision-making and enhances their sense of competence, thereby shifting exercise behavior from an external requirement to an intrinsic need (100). More importantly, the core intervention targets identified through network analysis—Intrinsic Motivation (IM) and Behavioral Habit (BH)—guide the design of efficient and cost-effective precision interventions. Practical efforts should focus on creating sports activities that stimulate students’ intrinsic interest and on using AI to support habit-forming training, thereby achieving a “small effort with big results” in promoting exercise. This provides concrete empirical evidence for implementing the Healthy China strategy and for the deep integration and innovative application of digital technologies in campus sports.

## 7 Limitations and future directions

This study has several limitations. First, the cross-sectional design fundamentally limits causal inference, as it provides only a snapshot of the variables at a single point in time. Consequently, the mediation model represents a theoretically informed associative structure rather than an empirically tested causal process. While the high centrality of intrinsic motivation and behavioral habits in the network analysis indicates meaningful connections, these centralities should not be interpreted as direct targets for intervention without further validation through more robust methodologies. Second, the stability coefficients for the centrality measures were only marginally above the acceptable threshold, which suggests that further research with additional validation techniques (e.g., longitudinal studies) could strengthen the reliability of these measures. A further limitation lies in the sampling method used for scale development, which focused on students with both regular exercise habits and prior experience with generative AI. While this was essential for ensuring a well-developed scale, it means the findings may not fully represent the broader student population, particularly those without established exercise routines or familiarity with AI. Future research should include a more diverse sample to assess the generalizability of the results.

Additionally, self-report data introduces the potential for common method bias. While Harman’s single-factor test provided a preliminary check (with the first factor explaining 21.24% of variance), this approach has recognized limitations and does not constitute a comprehensive assessment. Future studies could employ more robust procedural (e.g., temporal separation of measures and protection of respondent anonymity) and statistical remedies for CMB. Furthermore, integrating objective behavioral data (e.g., AI usage logs or fitness tracking data) would provide more comprehensive insights. Finally, this study touches upon important ethical and practical considerations regarding generative AI in health. The risk of AI “hallucinations”—where models generate plausible but incorrect or nonsensical exercise advice—represents a significant challenge for trust and safety. While not a focal point of the current survey, this inherent limitation of generative AI cannot be overlooked in practical applications. Such inaccuracies could undermine user trust, lead to ineffective or unsafe exercise practices, and raise concerns about accountability. This issue connects to broader debates on AI ethics in digital health, emphasizing the need for transparency about AI limitations, clear disclaimers, human oversight, and the development of more robust, verifiable systems. Future research should directly investigate how perceptions of AI reliability and transparency influence adoption intentions and trust, which are foundational for the ethical integration of these tools into health behavior interventions. Additionally, the use of measures with different Likert-scale formats (5-point *vs.* 7-point) is a methodological consideration, even though it is justified to maintain the validity of established scales. Future research could employ psychometric approaches, such as equating, or develop unified scaling to facilitate direct score comparisons across constructs.

## Data Availability

The original contributions presented in the study are included in the article/Supplementary material, further inquiries can be directed to the corresponding authors.
