# Supplementary material for: The impact of intention to adopt generative AI for exercise information on exercise adherence among Chinese college students: the mediating role of autonomous motivation and network analysis
Source: Front Public Health. 2026 Feb 13;14:1754288. doi: 10.3389/fpubh.2026.1754288 (PMC12946069; doi:10.3389/fpubh.2026.1754288)
Supplement: Supplementary file 1 [file Supplementary_file_1.docx]

Supplementary Material

# Supplementary Figures and Tables

## Supplementary Tables

Item analysis: We performed critical-ratio (CR) analysis and item–total correlation analysis, and removed items with CR < 3.0 or item–total correlation < 0.40. The results are presented in Table 1.

**Table S-1** Item analysis for the Generative AI Health Knowledge Acquisition scale.

| Dimension | items | t | r |
| --- | --- | --- | --- |
| Perceived Usefulness | PU1: I believe generative AI can provide me with scientific and effective exercise and fitness guidance. | -18.604 | .665*** |
|  | PU2: Using generative AI to obtain exercise information helps me achieve my exercise goals (such as muscle gain, fat loss, or improving fitness) more efficiently. | -19.344 | .655*** |
|  | PU3: Overall, I think using generative AI to assist with my exercise is very useful. | -20.976 | .674*** |
| Perceived Fit | PF1: I believe generative AI can generate "tailored" exercise plans based on my personal situation (such as fitness level, preferences, and available time). | -20.678 | .696*** |
|  | PF2: I trust that the exercise recommendations provided by generative AI will align well with my current exercise needs and goals. | -19.751 | .696*** |
|  | PF3: I think generative AI can flexibly adjust and optimize exercise plans based on my feedback (such as feeling too tired or lacking equipment). | -20.931 | .692*** |
| Attitude toward Use | ATU1: I believe using generative AI to obtain exercise information is a wise decision. | -18.115 | .656*** |
|  | ATU2: I enjoy using generative AI to help solve various problems I encounter during exercise. | -17.052 | .647*** |
|  | ATU3: For me, using generative AI to plan and manage my exercise is a pleasant experience. | -17.943 | .662*** |
| Adoption Intention | ADI1: I am willing to use generative AI frequently in the future to obtain exercise and fitness information and advice. | -17.569 | .662*** |
|  | ADI2: I plan to use generative AI as one of my primary tools for exercise information reference in future workouts. | -16.892 | .632*** |
|  | ADI3: I will recommend using generative AI to classmates or friends who have exercise needs. | -17.180 | .653*** |

Note: **p*< 0.05, ***p* < 0.01, ****p*< 0.001. The same notation applies below.

Internal consistency: The overall Cronbach’s a for the scale was 0.832. Cronbach’s a for each dimension are shown in Table 2.

**Table S-2** Summary of reliability analysis.

|  | **number of terms** | **Cronbach’s a** |
| --- | --- | --- |
| Perceived Usefulness | 3 | 0.924 |
| Perceived Fit | 3 | 0.931 |
| Attitude toward Use | 3 | 0.910 |
| Adoption Intention | 3 | 0.920 |
| Overall | 12 | 0.886 |

Exploratory Factor Analysis (EFA): Principal component analysis with varimax rotation was conducted. The KMO value exceeded 0.70, and Bartlett’s test of sphericity was significant (*p*< 0.001). Four common factors with eigenvalues greater than 1 were extracted, accounting for a cumulative variance of over 60%. All items had factor loadings above 0.55 with no cross-loadings. The results are shown in Table 4.

**Table S-3** KMO and Bartlett’s Test.

| Kaiser-Meyer-Olkin Measure of Sampling Adequacy. |  | 0.837 |
| --- | --- | --- |
| Bartlett's Test of Sphericity | Approx. Chi-Square | 4004.833 |
|  | df | 66 |
|  | Sig. | 0.000 |

**Table S-4** Rotated component matrix from exploratory factor analysis.

| Item | Factor | | | |
| --- | --- | --- | --- | --- |
|  | 1 | 2 | 3 | 4 |
| AI1 |  | 0.888 |  |  |
| AI2 |  | 0.913 |  |  |
| AI3 |  | 0.890 |  |  |
| AI4 | 0.887 |  |  |  |
| AI5 | 0.899 |  |  |  |
| AI6 | 0.902 |  |  |  |
| AI7 |  |  |  | 0.895 |
| AI8 |  |  |  | 0.875 |
| AI9 |  |  |  | 0.887 |
| AI10 |  |  | 0.901 |  |
| AI11 |  |  | 0.895 |  |
| AI12 |  |  | 0.900 |  |

Extraction Method: Principal Component Analysis.

Rotation Method: Varimax with Kaiser Normalization.

a Rotation converged in 5 iterations.

**Table S-5** Top 30 Correlation Coefficients between Nodes in the Network.

| Rank | Node |  | Node | Correlation Coefficient |
| --- | --- | --- | --- | --- |
| 1 | BH | - | EI | 0.252 |
| 2 | ATU | - | ADI | 0.250 |
| 3 | ER | - | IM | 0.235 |
| 4 | PU | - | ATU | 0.220 |
| 5 | IJR | - | IM | 0.208 |
| 6 | BH | - | AE | 0.207 |
| 7 | IJR | - | IDR | 0.202 |
| 8 | PF | - | ADI | 0.192 |
| 9 | PU | - | PF | 0.187 |
| 10 | ER | - | IJR | 0.183 |
| 11 | ER | - | IDR | 0.182 |
| 12 | EI | - | AE | 0.177 |
| 13 | IDR | - | IM | 0.176 |
| 14 | PF | - | ATU | 0.158 |
| 15 | PU | - | ADI | 0.115 |
| 16 | AE | - | IM | 0.085 |
| 17 | PF | - | AE | 0.080 |
| 18 | BH | - | ER | 0.078 |
| 19 | EI | - | IJR | 0.078 |
| 20 | ATU | - | BH | 0.066 |
| 21 | PU | - | ER | 0.064 |
| 22 | ADI | - | EI | 0.061 |
| 23 | PF | - | BH | 0.060 |
| 24 | AE | - | IJR | 0.057 |
| 25 | ATU | - | IM | 0.053 |
| 26 | EI | - | IDR | 0.053 |
| 27 | BH | - | IM | 0.052 |
| 28 | PU | - | BH | 0.049 |
| 29 | PU | - | AE | 0.048 |
| 30 | ADI | - | IDR | 0.046 |

**Table S-6** Centrality Scores of Nodes in the Network.

| Rank | Node | Expected Influence |
| --- | --- | --- |
| 1 | IM | 1.537 |
| 2 | BH | 1.245 |
| 3 | IJR | 0.553 |
| 4 | ER | 0.470 |
| 5 | ATU | 0.250 |
| 6 | ADI | -0.180 |
| 7 | IDR | -0.239 |
| 8 | PF | -0.239 |
| 9 | PU | -0.300 |
| 10 | AE | -1.064 |
| 11 | EI | -2.032 |

## Supplementary Figures

Confirmatory Factor Analysis (CFA): A three-factor model was constructed using AMOS (Figure 1). The fit indices ($\chi$^2^/df = 1.276, RMSEA = 0.026, SRMR = 0.036, CFI = 0.997, TLI = 0.995) indicated good model fit and reliable structural validity.


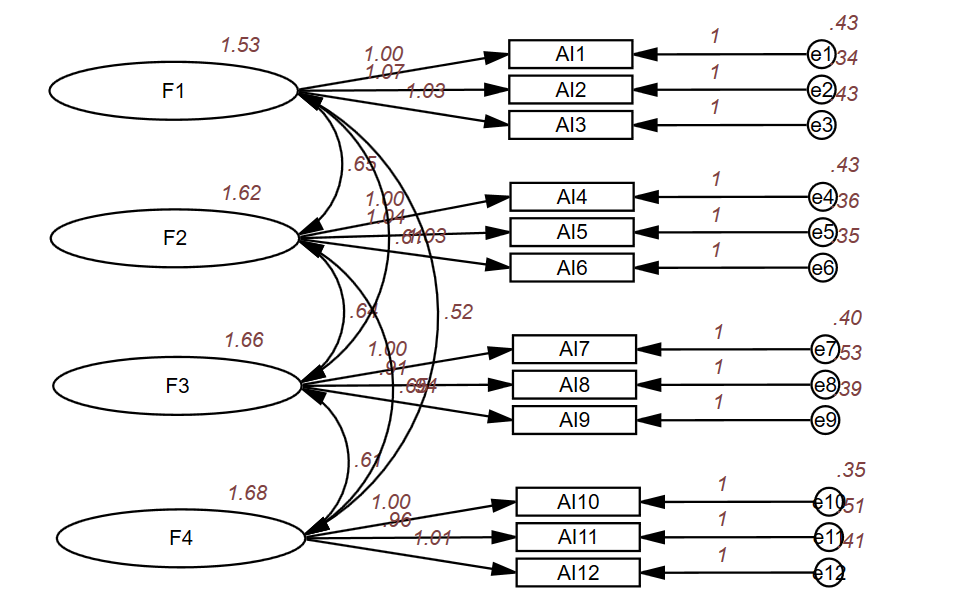


**Figure 1.** Confirmatory factor analysis model.


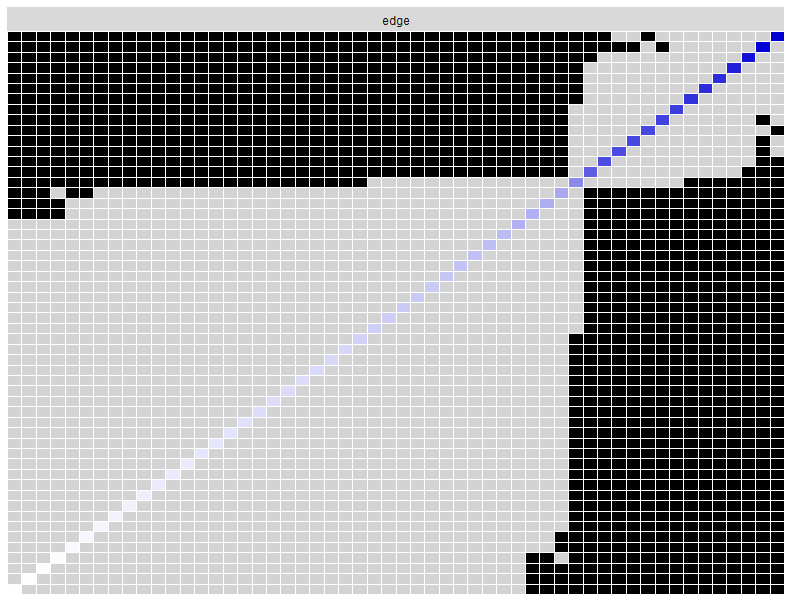


**Figure 2** Network Structure and Interaction between Variables.


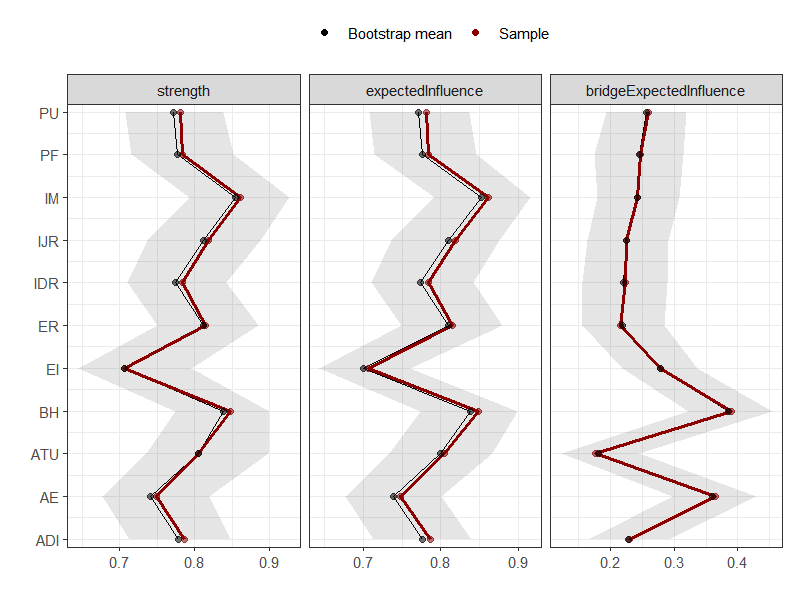


**Figure 3** Stability Test for Network Structure.


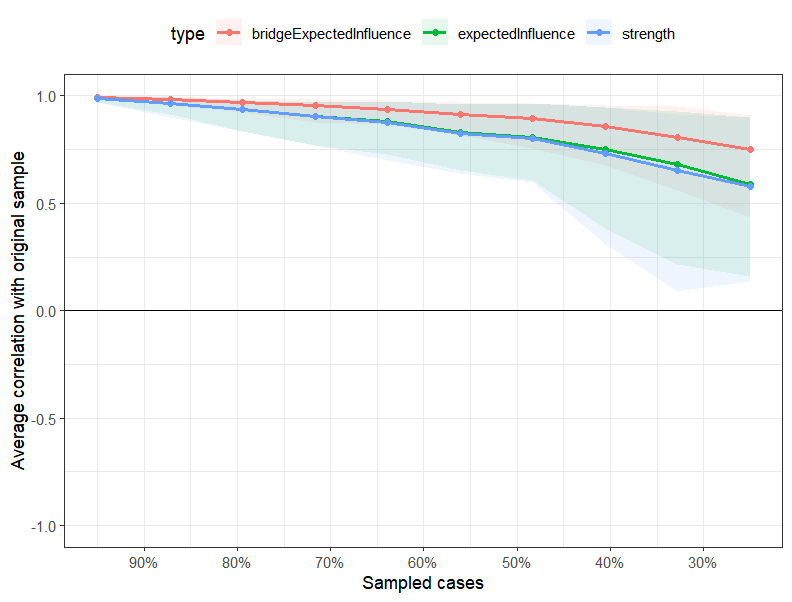


**Figure 4** Stability Test for Node Centrality.
